# Estimating acute human leptospirosis incidence in northern Tanzania using sentinel site and community behavioural surveillance

**DOI:** 10.1111/zph.12712

**Published:** 2020-05-06

**Authors:** Michael J. Maze, Katrina J. Sharples, Kathryn J. Allan, Holly M. Biggs, Shama Cash‐Goldwasser, Renee L. Galloway, William A. de Glanville, Jo E. B. Halliday, Rudovick R. Kazwala, Tito Kibona, Blandina T. Mmbaga, Venance P. Maro, Matthew P. Rubach, Sarah Cleaveland, John A. Crump

**Affiliations:** ^1^ Centre for International Health University of Otago Dunedin New Zealand; ^2^ Department of Medicine University of Otago Christchurch New Zealand; ^3^ Kilimanjaro Christian Medical Centre Moshi Tanzania; ^4^ Department of Mathematics and Statistics University of Otago Dunedin New Zealand; ^5^ Boyd Orr Centre for Population and Ecosystem Health Institute of Biodiversity, Animal Health and Comparative Medicine University of Glasgow Glasgow UK; ^6^ Division of Infectious Diseases Duke University Medical Center Durham NC USA; ^7^ Duke Global Health Institute Duke University Durham NC USA; ^8^ Bacterial Special Pathogens Branch Centers for Disease Control and Prevention Atlanta GA USA; ^9^ Department of Veterinary Medicine and Public Health Sokoine University of Agriculture Morogoro Tanzania; ^10^ Nelson Mandela African Institution for Science and Technology Arusha Tanzania; ^11^ Kilimanjaro Christian Medical University College Moshi Tanzania

**Keywords:** incidence, leptospirosis, risk factor

## Abstract

Many infectious diseases lack robust estimates of incidence from endemic areas, and extrapolating incidence when there are few locations with data remains a major challenge in burden of disease estimation. We sought to combine sentinel surveillance with community behavioural surveillance to estimate leptospirosis incidence. We administered a questionnaire gathering responses on established locally relevant leptospirosis risk factors and recent fever to livestock‐owning community members across six districts in northern Tanzania and applied a logistic regression model predicting leptospirosis risk on the basis of behavioural factors that had been previously developed among patients with fever in Moshi Municipal and Moshi Rural Districts. We aggregated probability of leptospirosis by district and estimated incidence in each district by standardizing probabilities to those previously estimated for Moshi Districts. We recruited 286 community participants: Hai District (*n* = 11), Longido District (59), Monduli District (56), Moshi Municipal District (103), Moshi Rural District (44) and Rombo District (13). The mean predicted probability of leptospirosis by district was Hai 0.029 (0.005, 0.095), Longido 0.071 (0.009, 0.235), Monduli 0.055 (0.009, 0.206), Moshi Rural 0.014 (0.002, 0.049), Moshi Municipal 0.015 (0.004, 0.048) and Rombo 0.031 (0.006, 0.121). We estimated the annual incidence (upper and lower bounds of estimate) per 100,000 people of human leptospirosis among livestock owners by district as Hai 35 (6, 114), Longido 85 (11, 282), Monduli 66 (11, 247), Moshi Rural 17 (2, 59), Moshi Municipal 18 (5, 58) and Rombo 47 (7, 145). Use of community behavioural surveillance may be a useful tool for extrapolating disease incidence beyond sentinel surveillance sites.

**Impacts**

- Leptospirosis, like many infectious diseases, lacks robust estimates of the number of cases that occur each year, particularly at areas that are not served by epidemiologic surveillance systems.
- We combined questionnaire data with a locally developed prediction model from a sentinel surveillance site to estimate the number of cases of leptospirosis in six districts across northern Tanzania.
- While our prediction model and our estimates contain considerable uncertainty, we think our method may have widespread use for leptospirosis and other infectious diseases.

## 1 INTRODUCTION

Our understanding of the burden of many infectious diseases in low‐resource areas is hampered by few robust estimates of incidence. The most rigorous approach to estimating incidence is through population‐based cohort studies. Population‐based approaches require substantial resources in order to recruit participants and maintain participation through the duration of the study (Szklo, 1998). Such studies have not been conducted for many infectious diseases in low‐resource areas, including leptospirosis.

In Tanzania, leptospirosis has been identified as prevalent among patients with fever (Crump et al., 2013), but there are few estimates of incidence (Allan et al., 2015; de Vries et al., 2014). We have previously estimated the annual leptospirosis incidence for Moshi Municipal District and Moshi Rural District, in the Kilimanjaro Region of Tanzania as 75–102 cases/100,000 people during 2007–2008 and 11–18 cases/100,000 people during 2012–2014 using multiplier studies that account for under‐ascertainment based on surveillance of acute leptospirosis among patients presenting to hospital (Biggs et al., 2013; Maze et al., 2016). However, Moshi Municipal District and Moshi Rural District are only two (1.2%) of 169 districts in Tanzania and tools are needed to infer disease incidence of leptospirosis away from sentinel surveillance sites across a broader geographical scope. Aside from our previous estimates of leptospirosis incidence in Moshi Districts, data on leptospirosis incidence are scarce. Costa and others estimated leptospirosis national incidence for Tanzania as 20.89 (95% confidence intervals [95% CI] 7.27, 38.34) as part of an estimate of global incidence. They used a prediction model that incorporated previously identified environmental and population risk factors for leptospirosis such as distance from the equator, percentage of the population urbanized, life expectancy at birth, and whether the country was a tropical island (Costa et al., 2015). While such estimates are useful, they focus on broad environmental risk factors, ignore the effects of human behaviour and do not address subnational variation. In addition, the authors acknowledged that their model was likely to be unreliable for African countries as the selected risk factors were derived almost exclusively from studies done elsewhere (Costa et al., 2015). In this context, we sought to estimate leptospirosis incidence in districts across northern Tanzania where surveillance data are lacking in order to better understand the variation in leptospirosis incidence at a subnational level.

Exposure to cattle and rodents has recently been identified as risk factors for human leptospirosis among patients with fever in northern Tanzania (Maze et al., 2018). Our case–control study used logistic regression to investigate associations between acute leptospirosis and scales of cumulative exposure to potential sources of infection that we identified from the published literature: urine of cattle, goats, pigs and rodents, and surface water (Ashford et al., 2000; Bovet, Yersin, Merien, Davis, & Perolat, 1999; Leal‐Castellanos, Garcia‐Suarez, Gonzalez‐Figueroa, Fuentes‐Allen, & Escobedo‐de la Penal, 2003; Mwachui, Crump, Hartskeerl, Zinsstag, & Hattendorf, 2015; Sarkar et al., 2002; Sugunan et al., 2009). The final multivariable model contained exposure to cattle urine (co‐efficient 0.16; 95% CI −0.05, 0.37) and exposure to rodent urine (co‐efficient 0.11; 95% CI −0.01, 0.25). We have also administered risk factor questionnaires to livestock‐owning community members as part of an ongoing study investigating risk factors for seroprevalence to bacterial zoonotic infections among livestock and their owners. We aimed to integrate hospital sentinel surveillance site data with data from community risk factor questionnaires to estimate the incidence of leptospirosis across a broad geographic area of northern Tanzania.

## 2 MATERIALS AND METHODS

### 2.1 Study setting

Arusha and Kilimanjaro regions are the two most populous regions of northern Tanzania. Each region is divided into seven districts. Human and animal population density, climate, and farming systems vary considerably between districts of the Arusha and Kilimanjaro regions with pastoralist farming predominating in Longido District and Monduli District, small‐holder farming predominating in Hai District, Moshi Rural District and Rombo District, and an urban environment predominating in Moshi Municipal District (2012 Population & Housing Census, 2013). Each district is subdivided into wards, each of which are further subdivided into villages.

### 2.2 Evaluation of leptospirosis prediction model among sentinel site patients with fever

The diagnostic accuracy of our previously developed leptospirosis risk factor multivariable model was evaluated among patients with fever in whom the model was developed. As reported previously (Maze et al., 2018), the patient population was recruited from paediatric and adult patients presenting with fever to two referral hospitals, Kilimanjaro Christian Medical Centre (KCMC) and Mawenzi Regional Referral Hospital (MRRH) in Moshi from 20 February 2012 through 28 May 2014. Participants provided acute serum and were requested to provide convalescent serum 4–6 weeks after enrolment. Serology for leptospirosis was performed on acute and convalescent serum samples using the standard microscopic agglutination test (MAT) with a panel of 20 *Leptospira* serovars belonging to 17 serogroups at the US Centers for Disease Control and Prevention. Leptospirosis cases were defined as participants with either a single reciprocal titre of ≥800 or a fourfold rise in titres between acute and convalescent serum samples (Centers for Disease Control & Prevention, 2013).

In our current study, we estimated the probability of leptospirosis among each fever study participant by using fitted values from the final multivariable model of acute leptospirosis, hereafter called the logistic regression model, to participants’ exposure scores. We evaluated errors associated with the magnitude of association of risk factors in our model by assessing the difference in estimated probability among cases and controls at the estimated upper and lower 95% confidence intervals of the co‐efficient for each risk factor. The diagnostic accuracy of the model was estimated by calculating the area under the receiver‐operator‐curve (AUROC). Out‐of‐sample error of the final exposure‐scale multivariable model was assessed using root mean square error (RMSE) evaluated through leave‐one‐out cross validation (Kohavi, 1995; Picard & Cook, 1984).

### 2.3 Cross‐sectional behavioural risk factor study among livestock‐keepers

We conducted a cross‐sectional study among livestock owners in the Arusha and Kilimanjaro regions from 4 September 2013 through 20 March 2015, using a multi‐stage random sampling process to select wards, villages, and households for sample visits. Sampling occurred within six districts: Longido District and Monduli Districts in Arusha Region, and Hai District, Moshi Rural District, Moshi Municipal District and Rombo District in Kilimanjaro Region.

#### 2.3.1 Behavioural surveillance

All members of selected households were approached for enrolment in the study. Trained study staff members who were fluent in the participant's language administered standardized questionnaires inquiring about established risk factors for zoonotic disease including leptospirosis from studies done in other settings (Ashford et al., 2000; Bovet et al., 1999; Leal‐Castellanos et al., 2003; Mwachui et al., 2015; Sarkar et al., 2002; Sugunan et al., 2009), adapted for the situation in northern Tanzania. We also asked whether fever had been present during the 2 weeks prior to the interview. The questionnaires were developed in conjunction with those administered at our sentinel sites at KCMC and MRRH to patients with fever (Maze et al., 2018) in order to harmonize analysis. Questionnaires were developed in English and translated by professional translators. Risk factors were aggregated into the scales of cumulative exposure to cattle urine and rodent urine that were analogous to the aggregated exposure scales developed among patients with fever at our sentinel hospital sites (Maze et al., 2018). Since questions on whether participants had fed cattle or worked in the sugarcane fields were not included in the questionnaire, there were minor differences in the weightings of those previously published (Table S1). We calculated a score, between 0 and 5 on each scale, for each participant, based on their questionnaire responses. A participant who had performed none of the exposure activities scored zero, and someone who performed all of the activities scored 5.

#### 2.3.2 Predicted probability of acute leptospirosis

We predicted the risk of leptospirosis during the 2 weeks prior to the interview for each participant in the cross‐sectional community dataset by applying the logistic regression model to participants who reported that they had experienced fever during the preceding 2 weeks. For those participants who did not report fever, we set the probability of leptospirosis during the 2 weeks prior to the interview as zero. We assessed the effect that plausible changes in the coefficient of the variables in our logistic regression model would have on the predicted probability of leptospirosis among participants by repeating probability predictions using the upper and lower bounds of the 95% confidence intervals of the regression coefficients.

#### 2.3.3 Prediction of incidence by district

We aggregated the predicted probability of recent leptospirosis for individuals by district and calculated the mean. We benchmarked predicted incidence in each District to that of Moshi Municipal District, where the leptospirosis incidence had been previously established as 11 cases per 100,000 people during the study period. To benchmark predicted incidence, we multiplied the incidence of leptospirosis in Moshi Municipal District by the ratio of the mean predicted probability of leptospirosis between the relevant district and Moshi Municipal District.

### 2.4 Data management

Data were entered using the Cardiff Teleform system (Cardiff, Inc.) into an Access database (Microsoft Corporation). Analyses were performed using Stata, version 13.1 (StataCorp).

### 2.5 Research ethics

This study obtained clearance from ethical review committees at KCMC, the National Institute of Medical Research (Tanzania), the University of Glasgow and the University of Otago, and an Institutional Review Board at Duke University.

## 3 RESULTS

### 3.1 Evaluation of leptospirosis prediction model among sentinel site patients with fever

**FIGURE 1:**
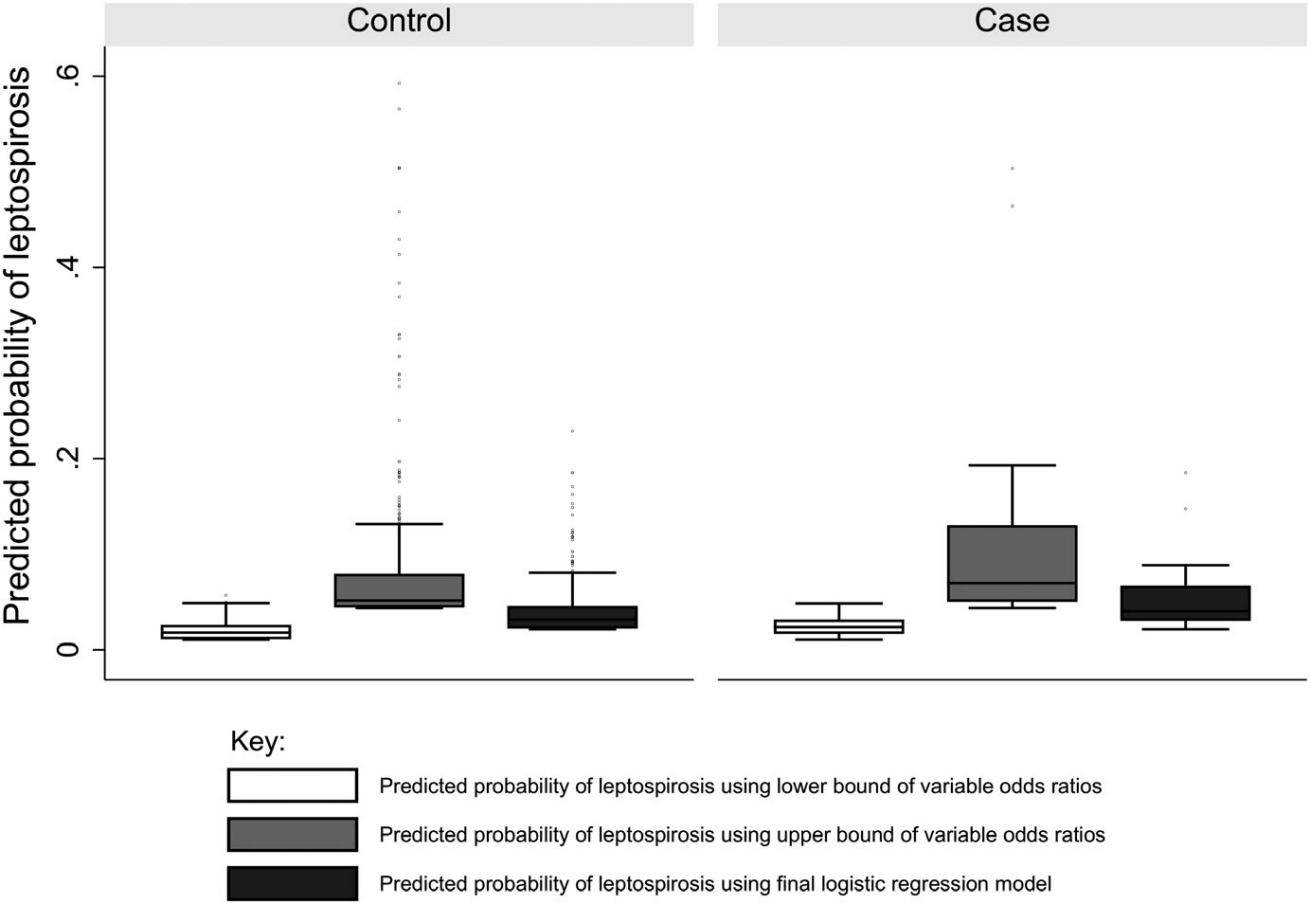
Predicted probabilities of leptospirosis among controls and leptospirosis cases in acute febrile illness study, northern Tanzania, 2012–2014

Figure 1 shows the distribution of predicted probabilities of leptospirosis that were obtained when using the point estimate and 95% confidence intervals of coefficients obtained from the final logistic regression model among leptospirosis cases (*n* = 24) and controls (*n* = 592). The distribution of probabilities was higher among cases than controls (Kruskal–Wallis test *p* value .01). The AUROC was 0.64. Leave‐one‐out cross‐validation among febrile patients of model found the RMSE = 0.193.

### 3.2 Cross‐sectional behavioural risk factor study among livestock keepers

We consented and administered questionnaires to 286 participants. The characteristics of participants, described by district, are summarized in Table 1. Of 280 who provided either a date of birth or an approximate age range, there were 19 (6.8%) individuals aged under 12 years, 184 (65.7%) aged from 12 through 55 years, and 77 (27.5%) aged >55 years. There were no identifiable differences in participant age structure by district. The proportions (95% CI) of participants reporting fever within the last 2 weeks in each district were Longido District 47.5 (34.2, 60.9) % and Monduli District 50.0 (36.3, 63.7) % in Arusha Region; Hai District 27.3 (6.0, 60.1) %, Moshi Rural District 13.6 (5.2, 27.4) % and Moshi Urban District 20.4 (13.1, 29.5) %, and Rombo District 30.8 (9.0%–61.4%) % in Kilimanjaro Region. The prevalence of individual potential risk factors for leptospirosis is shown in Table 2.

**TABLE 1:** Characteristics of cross‐sectional community study participants by District, northern Tanzania, 2013–2015

	Districts of Arusha Region				Districts of Kilimanjaro Region							
	Longido		Monduli		Hai		Moshi Rural		Moshi Municipal		Rombo	
	*N* = 59	% (95% CI)	*N* = 56	% (95% CI)	*N* = 11	% (95% CI)	*N* = 44	% (95% CI)	*N* = 92	% (95% CI)	*N* = 13 (%)	% (95% CI)
Age												
<12 years	3	5.1 (0.0, 10.6)	5	8.9 (1.5, 16.4)	0	0.0	2	10.5 (0.0, 10.7)	9	9.8 (3.7, 15.9)	0	0.0
12–55 years	45	76.2 (65.4, 87.1)	40	71.4 (59.6, 83.3)	6	54.5 (23.4, 83.3)	27	61.4 (47.0, 75.8)	58	63.0 (53.2, 72.9)	8	61.5 (35.1, 88.0)
>55 years	11	18.6 (8.7, 28.6)	8	14.3 (5.1, 23.5)	5	45.5 (16.7, 76.6)	14	31.8 (18.1, 45.6)	34	37.0 (27.1, 46.8)	5	38.5 (12.0, 64.9)

	*N* = 59		*N* = 56		*N* = 11		*N* = 44		*N* = 103		*N* = 13	
Tribe												
Chagga	0	0.0	0	0.0	11	100	32	72.7 (57.6, 84.0)	78	75.7 (66.4, 83.1)	13	100
Pare	0	0.0	0	0.0	0	0.0	3	6.8 (2.1, 19.4)	10	9.7 (5.3, 17.2)	0	0.0
Maasai	59	100.0	56	100	0	0.0	0	0.0	0	0.0	0	0.0
Sambaa	0	0.0	0	0.0	0	0.0	0	0.0	2	1.9 (0.5, 7.5)	0	0.0
Other	0	0.0	0	0.0	0	0.0	9	20.5 (10.9, 35.1)	13	12.6 (7.4, 20.6)	0	0.0
Education												
None	37	62.7 (49.6, 74.2)	37	66.1 (52.6, 7.73)	1	9.1 (11.3, 46.6)	4	9.1 (3.4, 22.1)	14	13.6 (8.2, 21.7)	2	15.4 (3.6, 46.7)
Primary	20	33.9 (22.9, 47.0)	18	32.1 (21.1, 45.5)	9	81.8 (47.3, 95.8)	34	77.3 (62.4, 87.4)	61	59.2 (49.4, 68.3)	8	61.5 (33.2, 83.7)
Secondary	1	1.7 (0.2, 19.4)	1	1.8 (0.2, 11.9)	1	9.1 (1.1, 46.6)	3	6.8 (2.2, 19.4)	20	19.4 (12.8, 28.2)	1	7.7 (0.9, 41.2)
High school	1	1.7 (0.2, 11.3)	0	0.0	0	0.0	0	0.0	2	1.9 (0.5, 7.5)	0	0.0
University	0	0.0	0	0.0	0	0.0	3	6.8 (2.1, 19.4)	6	5.8 (2.6, 12.4)	2	15.4 (3.6, 46.7)
Fever within last 2 weeks	28	47.5 (34.2, 60.9)	28	50.0 (36.3, 63.7)	3	27.3 (6.0, 60.1)	6	13.6 (5.2, 27.4)	21	20.4 (13.1, 29.5)	4	30.8 (9.0, 61.4)

	Mean (95% CI)		Mean (95% CI)		Mean (95% CI)		Mean (95% CI)		Mean (95% CI)		Mean (95% CI)
Stock numbers											
Number of cattle kept	38 (23, 70)		23 (15, 29)		2 (2, 3)		2 (0, 2)		0 (0, 2)		2 (1, 3)
Number of goats kept	62 (32, 129)		26 (16, 30)		0 (0, 2)		0 (0, 4)		4 (0, 8)		9 (6, 11)
Number of pigs kept	0 (0, 0)		0 (0, 0)		0 (0, 0)		0 (0, 1)		0 (0, 0)		0 (0, 0)

Abbreviations: CI, confidence intervals.

**TABLE 2:** Prevalence of cattle and rodent related risk factors for leptospirosis among cross‐sectional community study participants, by District, northern Tanzania, 2013–2015

Variable	Districts of Arusha Region						Districts of Kilimanjaro Region												Chi^2^ ^a^
	Longido *N* = 59			Monduli *N* = 56			Hai *N* = 11			Moshi Rural *N* = 44			Moshi Municipal *N* = 103			Rombo *N* = 13			
	*n*	%	95% CI	*n*	%	95 CI	*n*	%	95% CI	*n*	%	95% CI	*n*		95% CI	*n*	%	95% CI	*p*
Owned cattle	59	100	93.9, 100	56	100	93.6, 100	11	100	71.5, 100	29	65.9	50.1, 79.5	41	39.8	30.2, 49.9	10	76.9	46.4, 92.8	<.01
Milked cattle	27	45.8	32.7, 59.2	30	53.6	39.7, 67.0	7	63.6	30.8, 89.1	10	22.7	11.5, 37.8	13	12.6	6.9, 20.6	5	38.5	13.9, 68.4	<.01
Slept in the same room as cattle	3	5.1	10.6, 14.1	3	5.4	1.1, 14.9	2	18.2	2.3, 51.8	0	0.0	0.0, 8.0	1	1.0	0.0, 5.3	1	7.7	0.2, 36.0	.03
Cleaned up cattle waste	13	22.0	12.3, 34.7	13	23.2	13.0, 36.4	9	81.8	48.2, 97.7	23	52.3	36.7, 67.5	25	24.3	16.4, 33.7	6	46.2	19.2, 74.9	<.01
Herded cattle	38	35.6	23.6, 49.1	15	26.8	15.8, 40.3	0	0.0	0.0, 28.5	2	4.5	0.6, 15.5	6	5.8	2.2, 12.2	1	7.7	0.2, 36.0	<.01
Handled cattle placenta	14	23.7	12.3, 34.7	9	16.1	7.6, 28.3	2	18.2	2.3, 51.8	1	2.3	0.1, 12.0	9	8.7	4.0, 15.9	0	0.0	0.0, 24.7	<.01
Assisted with cattle birth	13	22.0	13.6, 36.6	10	17.9	8.9, 30.3	2	18.2	2.3, 51.8	1	2.3	0.1, 12.0	3	2.9	0.6, 8.3	2	15.4	1.9, 45.4	<.01
Handled aborted cattle products	4	6.8	1.8, 16.5	3	5.4	1.1, 14.9	0	0.0	0.0, 28.5	0	0.0	0.0, 8.0	0	0.0	0.0, 3.5	0	0.0	0.0, 24.7	.05
Slaughtered cattle	10	16.9	8.4, 29.0	9	16.1	7.6, 28.3	0	0.0	0.0, 28.5	2	4.5	0.6, 15.5	3	2.9	0.6, 8.3	1	7.7	0.2, 36.0	.01
Performed rodent control activities	47	79.7	67.2, 89.0	51	91.1	80.4, 97.0	9	81.8	47.3, 95.8	40	90.9	78.3, 97.4	94	91.3	84.1, 95.9	12	92.3	64.0, 99.8	.25
Handled rodent carcasses	4	6.8	1.8, 16.5	1	1.8	0.1, 9.6	0	0.0	0.0, 28.5	0	0.0	0.0, 8.0	3	2.9	0.6, 8.3	0	0.0	0.0, 24.7	.35
Disposed of rodent carcasses by feeding them to other animals	17	28.8	17.8, 42.0	18	32.1	20.2, 46.0	0	0.0	0.0, 28.5	0	0.0	0.0, 8.0	5	4.9	1.6, 11.0	0	0.0	0.0, 24.7	<.01
Saw rodents in the house	23	39.0	26.5, 52.6	6	10.7	4.0, 21.9	8	72.7	39.0, 94.0	14	31.8	18.6, 47.6	28	27.2	18.8, 36.8	4	30.8	9.1, 61.4	<.01
Saw evidence of rodents in the house	47	79.7	67.2, 89.0	49	87.5	75.9, 94.8	8	72.7	39.0, 94.0	24	54.5	38.8, 69.6	67	65.0	55.0, 74.2	12	92.3	64.0, 99.8	<.01
Saw evidence of rodents in the kitchen	14	23.7	13.6, 36.6	6	10.7	4.0, 21.9	7	63.6	30.8, 89.1	21	47.7	32.5, 63.3	30	29.1	20.6, 38.9	4	30.8	9.1, 61.4	<.01
Saw evidence of rodents in their compound	23	39.0	26.5, 52.6	20	35.7	23.3, 49.6	8	72.7	39.0, 94.0	22	50.0	34.6, 65.4	37	35.9	26.7, 46.0	8	61.5	31.6, 86.1	.07
Saw evidence of rodents in their fields	17	28.8	17.8, 42.0	35	62.5	48.5, 75.1	3	27.3	6.0, 61.0	24	54.5	38.8, 69.6	33	32.0	23.1, 42.0	3	23.1	5.0, 53.8	<.01

Abbreviation: CI, confidence intervals.

^a^ Chi^2^ refers to difference between districts.

In Longido District, Monduli District and Hai District, all participants owned cattle, whereas in Rombo District, 10 (76.9%, 95% CI, 46.4, 95.0%) of 13 participants owned cattle; in Moshi Rural District, 29 (65.9%, 95% CI, 50.1, 79.5%) of 44 participants owned cattle; and in Moshi Municipal District, 41 (39.8%, 95% CI, 30.2, 49.9%) of 102 participants owned cattle. Activities with a high risk of exposure to cattle urine such as birthing cattle and cleaning up cattle waste varied by district: 13 (22.0%, 95% CI, 12.3, 34.7) of 59 for each activity in Longido District, 10 (17.9%, 95% CI, 9.8, 30.3) and 13 (23.2%, 95% CI, 13.0, 36.4) for birthing cattle and cleaning cattle waste, respectively, in Monduli District, two (18.2%, 95% CI, 2.3, 51.8) and nine (81.1%, 95% CI, 48.2, 97.7) of 11 in Hai District, one (2.3%, 95% CI, 0.1, 12.0) and 23 (52.3%, 95% CI, 36.7, 67.5%) of 44 in Moshi Rural District, three (2.9%, 95% CI, 0.6, 8.3%) and 25 (24.3%, 95% CI, 16.4, 33.7%) of 103 in Moshi Municipal District and 0 (15.4%, 95% CI, 0.0, 24.7%) and six (46.2%, 95% CI, 19.2, 74.9) of 13 participants in Rombo District. There were differences in prevalence by district of the behaviours that conferred exposure to rodents. For example, participants saw rodents in their house in 23 (39%, 95% CI, 26.5, 52.6%) of 59 participants in Longido District, six (10.7%, 95% CI, 4.0, 21.9%) of 56 in Monduli District, eight (72.7%, 95% CI, 39.0, 94.0%) of 11 in Hai District, 14 (31.8, 95% CI, 18.6, 47.6%) of 44 in Moshi Rural District, 28 (27.2 95% CI, 18.8, 36.8%) of 103 participants in Moshi Municipal District and four (30.8%, 95% CI, 11.5, 60.4%) of 13 participants in Rombo District.

The mean estimated levels of exposure to cattle urine and rodent urine by district are shown in Table 3. The mean cattle exposure scores were higher in Longido District (1.1, 95% CI, 0.8, 1.3) and Monduli District (1.0, 95% CI, 0.8, 1.1) than in Moshi Municipal (0.4, 95% CI, 0.2, 0.5) and Moshi Rural Districts (0.5, 95% CI, 0.4, 0.7). There were no differences in the rodent exposure scores between districts.

**TABLE 3:** Mean estimated levels of exposure to leptospirosis sources, by District, among cross‐sectional community study participants, northern Tanzania, 2013–2015

Variable	Districts of Arusha Region		Districts of Kilimanjaro Region			
	Longido	Monduli	Hai	Moshi Rural	Moshi Municipal	Rombo
	Mean (95% CI)	Mean (95% CI)	Mean (95% CI)	Mean (95% CI)	Mean (95% CI)	Mean (95% CI)
Cattle urine exposure score	1.1 (0.8, 1.3)	1.0 (0.8, 1.1)	1.0 (0.6, 1.5)	0.5 (0.4, 0.7)	0.4 (0.2, 0.5)	0.6 (0.3, 0.8)
Rodent urine exposure score	1.8 (1.5, 2.0)	2.1 (2.0, 2.3)	2.3 (1.8, 2.8)	2.0 (1.8, 2.3)	2.0 (1.8, 2.1)	2.4 (1.9, 2.8)

### 3.3 Predicted probability of leptospirosis and estimation of incidence among livestock keepers

**FIGURE 2:**
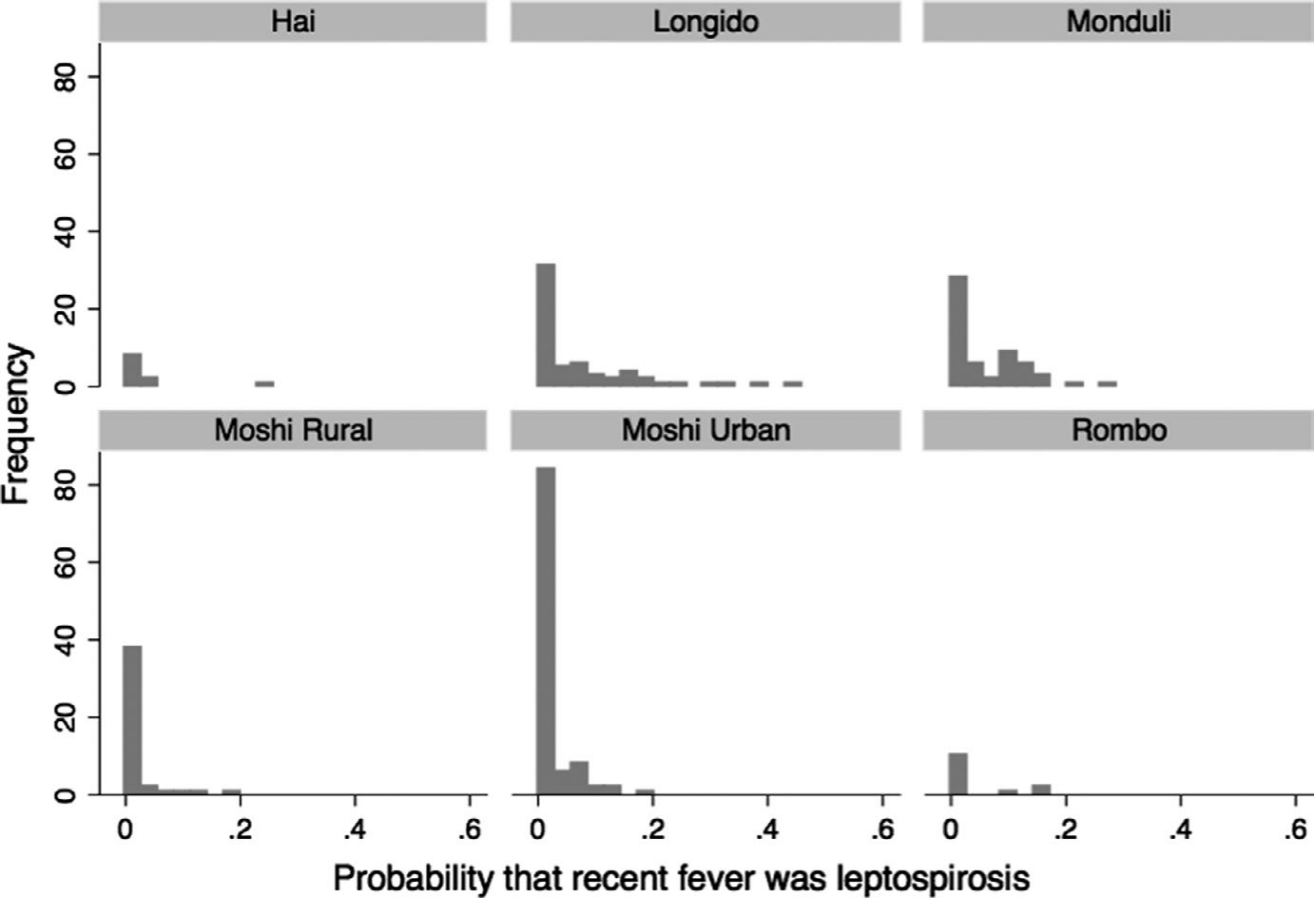
Histogram of predicted probability of leptospirosis during the preceding fortnight, among livestock keepers by District in northern Tanzania, 2013–2015

The mean predicted probability of leptospirosis in the previous 2 weeks among participants within each district, and the ratios of mean predicted probability in each district compared to Moshi Municipal District are shown in Table 4 and Figure 2. Leptospirosis annual incidence estimates are shown in Table 4. Participants in Moshi Rural District had the lowest predicted annual incidence of leptospirosis (10 cases per 100,000; upper and lower bounds 1, 36), and Longido District had the highest predicted annual incidence (53 cases per 100,000; upper and lower bounds 7, 174). When we performed a sensitivity analysis with Moshi Rural District as the benchmark, the estimated incidence ranged from 11 per 100,000 people in Moshi Rural District to 56 per 100,000 people in Longido District.

**TABLE 4:** Estimated incidence of leptospirosis among cross‐sectional community study participants, by District in northern Tanzania, 2013–2015

District	Mean predicted probability (upper, lower bounds)	Probability ratio	Annual leptospirosis incidence^a^ (upper, lower bounds)
Hai	0.029 (0.005, 0.095)	1.9	21 (4, 70)
Longido	0.071 (0.009, 0.235)	4.7	53 (7, 174)
Monduli	0.055 (0.009, 0.206)	3.7	41 (7, 153)
Moshi Rural	0.014 (0.002, 0.049)	0.9	10 (1, 36)
Moshi Municipal	0.015 (0.004, 0.048)	1.0	11 (3, 36)
Rombo	0.031 (0.006, 0.121)	2.1	23 (4, 90)

Note

Probability ratio = mean predicted probability/mean predicted probability in Moshi Municipal District.

^a^ Leptospirosis incidence set at 11 cases/100,000 people for Moshi Municipal District (Maze et al., 2016). The incidence in other districts estimated by multiplying Moshi Municipal District estimates by the probability ratio.

## 4 DISCUSSION

This study has applied and explored the limitations of a relatively simple method to estimate the incidence of leptospirosis across a broad area of northern Tanzania, including areas not served by leptospirosis surveillance. The predicted incidence of leptospirosis varied across the six districts from 10 to 53 cases per 100,000 people. The existing data used to estimate incidence had significant limitations and the estimates of districts overlap. We suggest that our numerical estimates should be viewed with caution as the risk factors for leptospirosis were modestly predictive. Despite these limitations, when behavioural risk factors are well defined, our approach may be useful for estimating the incidence of a range of infectious diseases in low‐resource areas that are not served by sentinel surveillance.

In many low‐ and middle‐income countries, our best estimates of incidence of infectious diseases come from studies that have use macro‐level risk factors to estimate incidence at a national scale, such as distance from the equator and per cent urbanization of the population (Costa et al., 2015; Mogasale et al., 2014). While such estimates are useful, they focus on broad environmental risk factors and ignore the effects of human behaviour. Our approach of estimating zoonotic disease incidence from locally relevant risk factors adds to the country‐level environmental risk factor approach and provides a useful tool for extrapolating data from sentinel sites across broad subnational areas. Our study aimed to develop novel methods of estimating incidence, and an important next step is to validate our approach by estimating leptospirosis incidence using more established methods in the districts studied here.

Our probability estimates rely on the assumption that the risk factors measured adequately account for leptospirosis risk, that the risk factors operate consistently between districts and that the proportion of fevers caused by leptospirosis is similar among community members reporting fever and hospitalized patients reporting fever. To mitigate these assumptions, we have assessed the potential for error at each stage of the modelling process and estimated confidence intervals to account for each potential error. Based on the AUROC and RMSE, our model is an imperfect predictor of disease, limiting its use for out‐of‐sample datasets. The wide range between the upper and lower bounds of our probability estimates reflects the poor prediction and uncertainty of our model. Our focus on behavioural risk factors does not account for variation in *Leptospira* shedding by reservoir hosts nor variation in *Leptospira* environmental persistence by location. There is currently lack of sufficient data on the prevalence of *Leptospira* shedding by livestock and wildlife hosts, and on the prevalence of *Leptospira* in waterways and soil in northern Tanzania to incorporate it in a prediction model. Such data will be important to improve accuracy of leptospirosis incidence estimation.

The highest point estimates of incidence were in Longido District and Monduli District, although the lower bounds of the incidence estimates overlapped the upper bounds of leptospirosis incidence estimated for Moshi Municipal District and Moshi Rural District. The higher point incidence estimates were driven by variation in the prevalence of fever and the increased exposure to livestock. Recent symptoms of fever were more commonly reported by participants from Longido and Monduli Districts than by participants from Moshi Rural and Moshi Municipal Districts. This finding is in keeping with previous reports (Lawson et al., 2014) and suggests a higher incidence of infectious disease associated with fever in those districts. In Moshi Rural and Moshi Urban Districts, major causes of fever include bloodstream infections, notably typhoidal and non‐typhoidal *Salmonella enterica,* bacterial zoonotic infections (i.e. brucellosis, leptospirosis, Q fever, spotted fever group rickettsiosis) and arboviral infections (Crump et al., 2013). Malaria is unlikely to account for differences in fever prevalence as the prevalence of *Plasmodium* parasitemia in all study districts is low (Hochedez et al., 2015). The higher prevalence of behaviours exposing participants to livestock in Longido and Monduli Districts compared with Moshi Rural District and Moshi Municipal District suggests an increased prevalence of zoonotic diseases, including leptospirosis, might contribute to the higher prevalence of reported fever. An alternative explanation for an increased prevalence of reported fever is a different cultural interpretation of the term ‘fever’ among those living in Longido District and Monduli District. Although previous work has indicated that biomedical terms are in common use among Maasai in northern Tanzania (Queenan et al., 2017), the low educational level of participants in Longido District and Monduli District may also have affected their health literacy and understanding of the term. Variation in understanding of biomedical terms among Maasai participants might be mitigated in future studies by ethnic Maasai research staff conducting interviews among Maasai participants.

Our use of data from the cross‐sectional study has a number of limitations that influence interpretation. Our study enrolled only livestock owners and did not seek to determine the proportion of the population that owned livestock. Therefore, we are uncertain of the application of findings to the non‐livestock owning population. Given that the number of livestock, and the ratio of livestock to people in both Longido and Monduli districts is higher than in Moshi Rural and Moshi Urban districts (2012 Population & Housing Census, 2013), we might expect that difference in prevalence of risk factors is in fact greater than we have observed. The size of the population sampled in Hai and Rombo districts was small. The uncertainty estimates are correspondingly large and limit interpretation of results from these districts.

Future attempts to estimate leptospirosis incidence using our approach require a model with greater predictive power. Additional factors that would improve the predictive accuracy of the model include distal risk factors such as used by Costa and colleagues in their global estimates of incidence (Costa et al., 2015), as well as data on the prevalence of *Leptospira* in soil, waterways and animal hosts. The parent study from which our data were collected will provide data on livestock and human *Leptospira* seroprevalence. While the complexities of the relationship between *Leptospira* seropositivity and acute leptospirosis infections make determination of incidence solely through seroprevalence challenging (Cumberland, Everard, Wheeler, & Levett, 2001; Haake & Levett, 2015), seroprevalence data could provide additional supporting evidence to risk factor‐based estimates of incidence. As well as improving our prediction model, future studies that estimate disease incidence from risk factor surveillance should include representative sampling of the entire population.

In conclusion, we extrapolated existing estimates of leptospirosis incidence across a broad geographical area using behavioural surveillance. While our approach could be improved through further data collection to develop a prediction model with greater accuracy, we propose that our approach may have application across many infectious diseases in low‐resource areas.

## CONFLICT OF INTEREST

The authors report no conflict of interest.

## DISCLAIMER

The findings and conclusions in this report are those of the authors and do not necessarily represent the official position of the Centers for Disease Control and Prevention. Use of trade names and commercial sources is for identification only and does not imply endorsement by the US Department of Health and Human Services or the Centers for Disease Control and Prevention.

## Supporting information

TableS1
