# Supplementary material for: Estimating acute human leptospirosis incidence in northern Tanzania using sentinel site and community behavioural surveillance
Source: Zoonoses Public Health. 2020 May 6;67(5):496–505. doi: 10.1111/zph.12712 (PMC7497209; doi:10.1111/zph.12712)
Supplement: Supplementary file 1 — TableS1 [file ZPH-67-496-s001.docx]

**Supplementary Table 1.** **Component risk factors and relative weights for exposure to cattle urine and rodent urine derived from an analytic hierarchy process conducted among East African subject matter experts, 2015**

| **Cattle urine exposure** | | **Rodent urine exposure** | |
| --- | --- | --- | --- |
| Variable | Weight | Variable | Weight |
| Clean livestock waste | 0.92 | Subsistence farmer | 0.70 |
| Birth livestock | 0.81 | Handle rat carcasses | 0.91 |
| Keep livestock inside house | 0.76 | See rats in kitchen | 0.77 |
| Milk livestock | 0.76 | Plumber | 0.72 |
| Slaughter livestock | 0.67 | See rats in house | 0.66 |
| Veterinarian | 0.54 | Kill rats | 0.63 |
| Herd livestock | 0.28 | See evidence of rats in house | 0.56 |
| Keep livestock around house | 0.27 | See evidence of rats in compound | 0.38 |
|  |  | See evidence of rats in fields | 0.37 |
| Total | 5.00 |  | 5.00 |
